# SMART: recent updates, new developments and status in 2015

**DOI:** 10.1093/nar/gku949

**Published:** 2014-10-09

**Authors:** Ivica Letunic, Tobias Doerks, Peer Bork

**Affiliations:** 1 Biobyte solutions GmbH, Bothestr 142, 69126 Heidelberg, Germany; 2 EMBL, Meyerhofstrasse 1, 69117 Heidelberg, Germany

## Abstract

SMART (Simple Modular Architecture Research Tool) is a web resource (http://smart.embl.de/) providing simple identification and extensive annotation of protein domains and the exploration of protein domain architectures. In the current version, SMART contains manually curated models for more than 1200 protein domains, with ∼200 new models since our last update article. The underlying protein databases were synchronized with UniProt, Ensembl and STRING, bringing the total number of annotated domains and other protein features above 100 million. SMART's ‘Genomic’ mode, which annotates proteins from completely sequenced genomes was greatly expanded and now includes 2031 species, compared to 1133 in the previous release. SMART analysis results pages have been completely redesigned and include links to several new information sources. A new, vector-based display engine has been developed for protein schematics in SMART, which can also be exported as high-resolution bitmap images for easy inclusion into other documents. Taxonomic tree displays in SMART have been significantly improved, and can be easily navigated using the integrated search engine.

## INTRODUCTION

Protein domain analysis remains an important research tool, made easy by various frequently used online domain resources and databases (e.g. 1–3). The SMART database (4) integrates manually curated hidden Markov models (5) for many domains with a powerful web-based interface offering various analysis and visualization tools. After more than 15 years since its inception it remains a popular and widely used by scientists worldwide. Here we give an overview of the major developments and new features introduced since our last update (6).

## EXPANDED DOMAIN COVERAGE

SMART was never intended to be exhaustive, and was initially focused on mobile domains, which occur in various contexts while retaining similar function. Nevertheless, it continues to gradually expand its domain coverage with each new release. The current version introduces more than 200 new domains, bringing the total to 1204 distinct modules that can be detected. SMART's domain annotation includes a significant amount of manual work, in particular when selecting individual cut-off values and while creating the high quality underlying multiple sequence alignments. Other more exhaustive databases, like Pfam (3), already annotated many of these domains, but SMART's own manual annotation pipeline leads to partially different protein annotations, enabling increased hypothesis generation by biologists.

## UPDATED PROTEIN DATABASES

The main protein database in SMART consists of the complete UniProt protein database (7) combined with predicted proteins from all stable Ensembl (8) genomes. The latest update greatly expanded the size of the database, which now contains more than 33 million proteins from around 350 000 species, subspecies and varietas. To minimize the impact of the inherently high redundancy of these databases, we use a per-species clustering method described in (9), which created 1.3 million multiprotein clusters with a total of 2.7 million proteins.

In addition to the regular protein database described above, SMART offers a ‘genomic’ analysis mode that contains only proteins from completely sequenced genomes. Synchronized with the upcoming STRING version 10 (10), this database has also been significantly extended, and currently contains ∼9.6 million proteins from 2031 complete genomes (238 *Eukaryota*, 1678 *Bacteria* and 115 *Archaea*), compared to 5 million proteins from 1133 species in the previous release.

## REDESIGNED PROTEIN ANNOTATION PAGES

**Figure 1.**
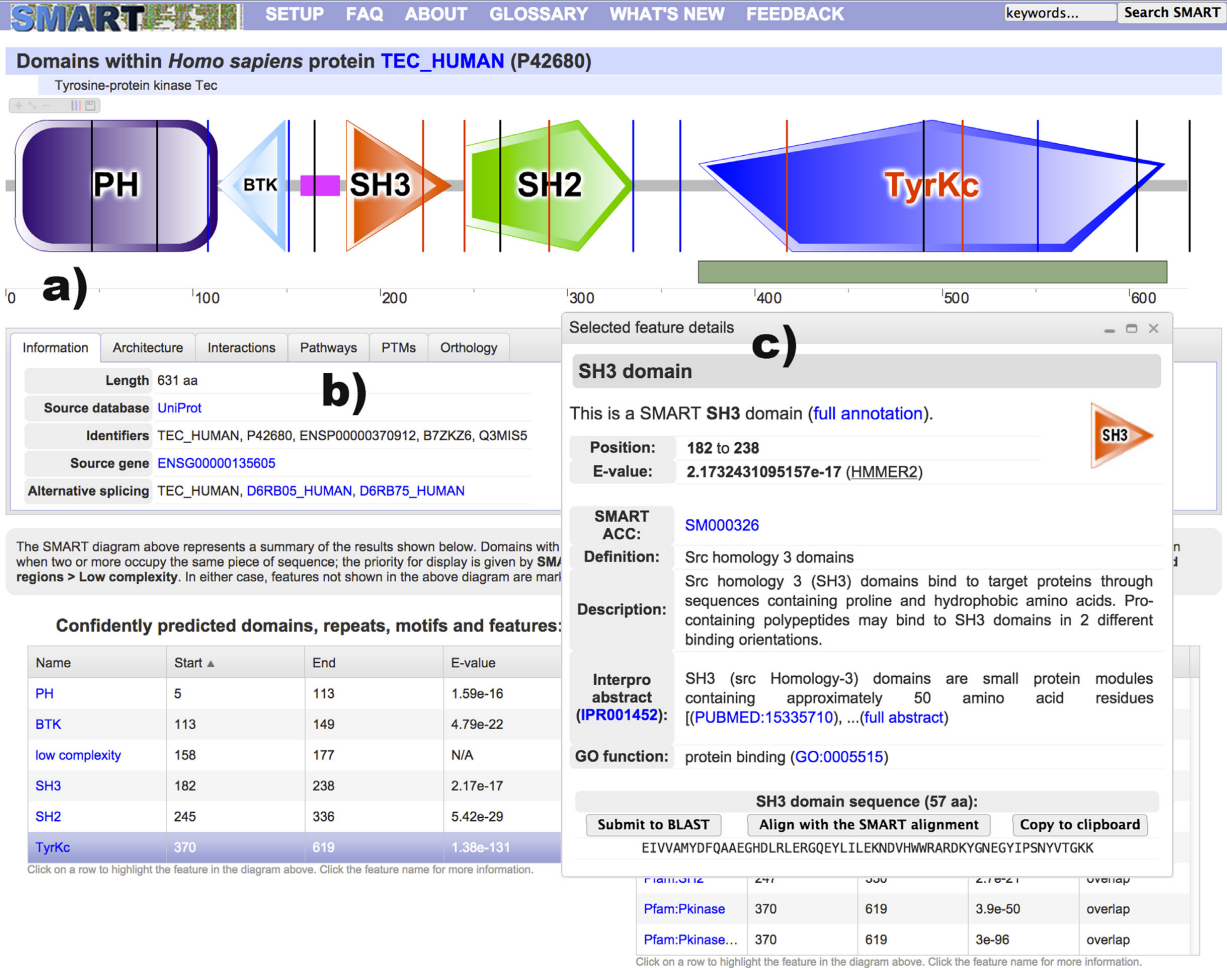
SMART annotation page for protein TEC_HUMAN. **(a)** Protein schematic representations are displayed using an interactive Flash applet. Schematics are zoomable without quality loss and exportable into high resolution bitmap images. Protein features selected in various data tables are dynamically highlighted directly in the viewer. Using the interactive scale, any protein region can be selected and submitted for further BLAST analysis. **(b)** The tabbed interface collects various sources of external information about the protein analyzed. **(c)** A movable and resizable popup dialog displays the most important bits of information for any selected feature, with links to complete annotation.

The current SMART version introduces completely re-designed protein annotation pages, with a new, vector-based protein schematic display engine (Figure 1). SMART protein schematics (‘bubblograms’) are now drawn using an Adobe Flash-based applet, greatly improving user experience. Schematics can be zoomed and exported into high resolution bitmap images. A function box within the interactive viewer provides access to several additional functions, for example, allowing users to toggle the display of intron positions or to navigate among various alternative representations of proteins containing overlapping domain predictions.

The protein viewer is connected to various parts of the annotation page. Selecting a predicted domain or other feature in any of the data tables (Figure 1) will automatically highlight its position in the protein. This is particularly useful for features that are not directly displayed in the protein schematic, either because they overlap other predicted domains or they get excluded due to E-value cutoffs.

Detailed information about any detected protein feature can be displayed in a simple floating popup dialog, streamlining the user experience and lowering the need to navigate across different web pages. In addition, these include several convenience functions, allowing users to copy the underlying amino acid sequence to their clipboard, or to submit the sequence for further Basic Local Alignment Search Tool (BLAST) analysis. These information dialogs contain condensed versions of respective annotation pages, with links to the full annotation. The new viewer also includes an interactive protein size scale, which allows users to directly select any protein region and submit it to a BLAST service of choice.

## EXPANDED AND UPDATED EXTERNAL INFORMATION SOURCES

**Figure 2.**
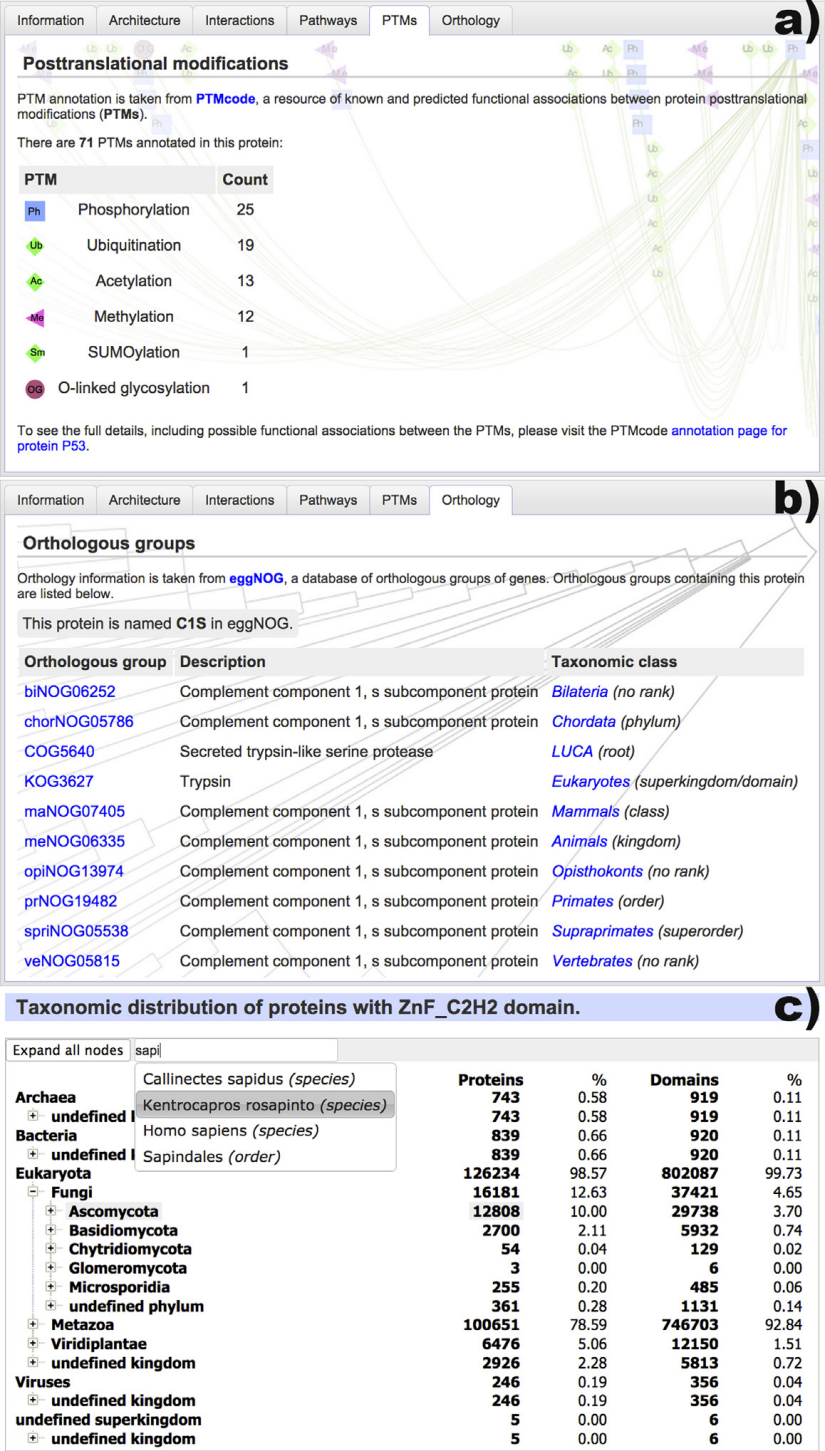
New data sources included in the protein annotation pages and the redesigned taxonomy viewer. (a) A list of post-translational modifications present in the protein, as annotated by PTMcode (11). More than 60 types of modifications are included. (b) Orthologous groups that contain the protein, as annotated by eggNOG (12). Group descriptions and taxonomic classes are listed. (c) New taxonomic breakdown viewer, which supports very large trees and provides quick navigation through the integrated full text search engine.

With the redesign of the protein annotation pages, the current version of SMART introduces two new external information sources: post-translational modification data and detailed orthology information (Figure 2).

Data on post-translational protein modifications is provided by version 2 of the PTMcode database (11), and is available for almost 400 000 proteins. SMART displays the total numbers of various post-translational modifications annotated in a particular protein, with links to the detailed annotation pages in PTMcode, where users can explore the modifications, their possible functional associations and the reasoning for calling them in detail.

Protein orthology data are parsed from the eggNOG database (12) and cover more than 7.7 million proteins from 3686 species. SMART's annotation pages show a detailed list of all orthologous groups that include the protein annotated, with their description and taxonomic class. Crosslinks to eggNOG are provided, with detailed overviews of each orthologous group as well as the associated alignments and phylogenetic trees.

## EXPANDED BIOLOGICAL PATHWAY AND PROTEIN INTERACTION DATA

With the update of the underlying protein databases, we have also synchronized our protein interaction data with the latest version of the STRING database (10). Graphical representations of putative interaction partners are now available for more than 9.5 million proteins, which is a 3-fold increase from the previous release.

SMART's integration of biological pathways data was greatly expanded in the current version, and is now synchronized with version 2 of the interactive Pathways Explorer (iPath2) (13). Available for more than 2 million proteins, the pathway information now includes not only links to metabolic pathways, but also a selection of regulatory pathways and a large set of pathways involved in the biosynthesis of secondary metabolites.

Biological pathway data, protein interaction data and their associated graphical representations are now part of SMART's new tabbed annotation interface (Figure 1), displaying only subsets of the information as requested by users, making navigation simpler and more user-friendly.

## UPDATED TAXONOMIC TREE DISPLAYS

SMART uses simple tree structures to display various taxonomic breakdowns in different parts of its user interface. For example, these are used to show the evolutionary information in domain annotation pages or to display the taxonomic breakdown of domain architecture queries. Since our current database contains proteins from more than 350 000 species, we developed a new tree display widget with an associated full text search engine (Figure 2c). It supports extremely large trees, which can still be navigated with ease. In addition, evolutionary breakdowns in the domain annotation pages now include both protein and domain counts for each taxonomic class, providing a much better overview for various domains that commonly occur in multiple copies per protein.

## BACKEND OPTIMIZATIONS

The backend of SMART is a relational database management system, powered by the PostgreSQL engine, which stores the annotation of all SMART domains, protein annotation and sequences, taxonomy information and the pre-calculated protein analyses for the entire UniProt (7), Ensembl (8) and STRING (10) sequence databases. This includes the predictions of SMART and Pfam domains, as well as various protein intrinsic features, like signal peptides, transmembrane and coiled coil regions. Our last update expanded the number of annotated domains and other protein features to more than 100 million, which caused significant slowdowns in various domain architecture analysis queries and made it necessary to restructure significant portions of the database, and to rewrite many parts of the backend code.
